# Developing expressed sequence tag libraries and the discovery of simple sequence repeat markers for two species of raspberry (*Rubus* L.)

**DOI:** 10.1186/s12870-015-0629-8

**Published:** 2015-10-26

**Authors:** Jill M. Bushakra, Kim S. Lewers, Margaret E. Staton, Tetyana Zhebentyayeva, Christopher A. Saski

**Affiliations:** USDA-ARS, National Clonal Germplasm Repository, 33447 Peoria Road, Corvallis, OR 97333-2521 USA; USDA-ARS, Beltsville Agricultural Research Center, Genetic Improvement of Fruits and Vegetables Lab, Bldg. 010A, BARC-West, 10300 Baltimore Ave., Beltsville, MD 20705-2350 USA; Department of Entomology and Plant Pathology, University of Tennessee, 2505 EJ Chapman Drive, 370 PBB, Knoxville, TN 37996 USA; Genomics & Computational Biology Laboratory, Biosystems Research Complex, Clemson University, 51 New Cherry St., 304, Clemson, SC 29634 USA

**Keywords:** Molecular markers, EST-SSR, *Rubus idaeus*, *Rubus occidentalis*, Microsatellites, Marker-assisted breeding, Marker transferability

## Abstract

**Background:**

Due to a relatively high level of codominant inheritance and transferability within and among taxonomic groups, simple sequence repeat (SSR) markers are important elements in comparative mapping and delineation of genomic regions associated with traits of economic importance. Expressed sequence tags (ESTs) are a source of SSRs that can be used to develop markers to facilitate plant breeding and for more basic research across genera and higher plant orders.

**Methods:**

Leaf and meristem tissue from ‘Heritage’ red raspberry (*Rubus idaeus*) and ‘Bristol’ black raspberry (*R. occidentalis*) were utilized for RNA extraction. After conversion to cDNA and library construction, ESTs were sequenced, quality verified, assembled and scanned for SSRs.  Primers flanking the SSRs were designed and a subset tested for amplification, polymorphism and transferability across species. ESTs containing SSRs were functionally annotated using the GenBank non-redundant (nr) database and further classified using the gene ontology database.

**Results:**

To accelerate development of EST-SSRs in the genus *Rubus* (Rosaceae), 1149 and 2358 cDNA sequences were generated from red raspberry and black raspberry, respectively. The cDNA sequences were screened using rigorous filtering criteria which resulted in the identification of 121 and 257 SSR loci for red and black raspberry, respectively. Primers were designed from the surrounding sequences resulting in 131 and 288 primer pairs, respectively, as some sequences contained more than one SSR locus. Sequence analysis revealed that the SSR-containing genes span a diversity of functions and share more sequence identity with strawberry genes than with other Rosaceous species.

**Conclusion:**

This resource of *Rubus*-specific, gene-derived markers will facilitate the construction of linkage maps composed of transferable markers for studying and manipulating important traits in this economically important genus.

**Electronic supplementary material:**

The online version of this article (doi:10.1186/s12870-015-0629-8) contains supplementary material, which is available to authorized users.

## Background

Red raspberry (*Rubus idaeus* L.) is an important fruit crop grown world-wide in the Northern and Southern hemispheres; black raspberry (*R. occidentalis* L.) is a specialty crop grown mainly in the Pacific Northwest of the United States. Interest in improvement of these crops is increasing in light of studies on their nutritional and nutraceutical value [1–4]. Development of new cultivars can benefit from reliable markers linked to important traits, including disease resistance, flowering traits, fruit quality characteristics, and plant architecture. Because interspecific hybridization was widely used by caneberry breeders [5, 6], markers that are transferrable between black and red raspberry and even between raspberry and blackberry would be especially useful. In addition, transferable *Rubus* markers could further illuminate mechanisms of sub-genomic organization in hybrids between disomic and polysomic species [7, 8]. Very few molecular markers exist for *Rubus* in general [9–12] and fewer are transferable between species [10, 13–15]. Several genetic linkage maps composed of various types of molecular markers are available for raspberry [14, 16–19], and one is available for blackberry [12], however, not all marker types used to construct these maps are transferable between taxa. Many more *Rubus* molecular markers and other genomic tools are needed to map important traits, facilitate cultivar development, maintain cultivar identity, and study basic genetic and genomic mechanisms.

Molecular markers designed from simple sequence repeats (SSR), tandem repeats of 1–6 nucleotides that frequently show co-dominant inheritance, are known to be highly variable even within species, and are transferable across taxa to a varying extent [20]. Gene-based SSR loci derived from expressed sequence tag (EST-SSR) are significantly more transferable across large taxonomic distances compared with genomic SSRs [21]. This feature makes EST-SSRs superior for comparative linkage mapping and interspecific cross-verification and manipulation of genomic regions associated with phenotypic traits [11, 18, 22–30]. However, EST resources available for the genus *Rubus* at the National Center for Biotechnology Information’s (NCBI) GenBank are scarce with only 3184 and 50 cDNA sequences for *R. idaeus* and *R. occidentalis,* respectively (accessed on January 24, 2015). A main impetus for this sequencing project was to generate a useful set of EST-SSR markers to enable further genetic research into the raspberry genome, and to increase the number of DNA sequences available for the Rosaceae research community and raspberry breeders. EST-SSRs reported here can significantly advance comparative linkage analysis among *Rubus* species.

## Results and discussion

### Red raspberry cDNA library construction and SSR discovery

A red raspberry cDNA library of 18,432 clones (48 plates in a 384-well format) was produced from *Rubus idaeus* cv. Heritage [31]. ‘Heritage’ is a widely grown, everbearing cultivar with resistance to most common raspberry diseases, and medium to large sized fruit with good color, flavor, firmness and freezing quality [32]. The cDNA library was prepared from the newly emerging leaves of a single plant. A cDNA library subset consisting of 1824 clones was sequenced with Sanger technology [33] (Clemson University Genomics & Computational Biology Laboratory, Clemson, SC, USA) yielding 1149 high quality sequences after removal of sequence shorter than 100 base pairs (bp) reported as accession numbers JZ840520 through JZ841668 in GenBank. The resulting sequences had an average length of 429 bp and an average Phred quality score [34] of 48. Transcripts derived from the same expressed gene sequence were assembled into 136 contiguous sequences (contigs) and 732 singletons, yielding a unique gene sequence or “unigene” of 868 sequences, thus reducing locus redundancy and inflation of marker numbers derived from a single locus.

A search for SSR loci within the unigenes using the SSR mining script tool found in the Toolbox on the Genome Database for Rosaceae [35, 36] identified 121 short, perfect repeats in the unigene sequences, which are candidate regions for high polymorphism. Trimers, 3 bp repeats, are more common repeat lengths for gene coding regions, likely because their increase or decrease in repeat number does not cause a reading frame shift [37]. This dataset did demonstrate this tendency with 30 % dimers (2 bp repeat motif), 44 % trimers (3 bp repeat motif), 20 % tetramers (4 bp repeat motif) and 6 % pentamers (5 bp repeat motif). Primers were designed to facilitate the amplification of the SSR loci, yielding 131 primer pairs suitable for testing 98 individual unigenes (Additional file 1).

### Black raspberry cDNA library construction and SSR discovery

*Rubus occidentalis* cv. Bristol [38] was chosen for construction of the black raspberry transcript library. ‘Bristol’ fruit ripen early, are medium sized and firm with excellent flavor; plants are susceptible to anthracnose and tolerant to powdery mildew [39]. The cDNA library was prepared from the newly emerging leaves of a single plant. The same number of cDNA clones was produced as for ‘Heritage’, 18,432. Because of expected low polymorphism rate in black raspberry [40–42], 4032 clones were sequenced with a final yield of 2358 high quality sequences after quality control analysis, reported as accession numbers JZ841669 through JZ844026 in GenBank. These sequences averaged 523 bp with an average Phred score of 50. The assembly consisted of 1422 unigenes (273 contigs, 1149 singletons).

A total of 257 SSR sequences were identified and showed a very similar composition to the red raspberry motif lengths: 35 % dimers, 40 % trimers, 21 % tetramers and 5 % pentamers. The final set of 288 primer pairs covers 207 unigenes (Additional file 2).

The percentages of each motif are generally as expected in plants [43, 44], and a high percentage of tetramers is not uncommon in plants [35]. An elevated number of tetramer repeats is thought to be an indication that the majority of this motif length may be found in non-coding regions of the expressed genes [43].

### Amplification using designed primer pairs

A random selection of SSR loci was tested for PCR amplification, amplification of a polymorphic PCR product, and transferability between species. A subset of 36 primer pairs from the 131 designed to test 98 individual unigenes identified in red raspberry, and 24 primer pairs from the 288 designed to test 207 unigenes identified in black raspberry were assessed using two genotypes each of *R. idaeus* (‘Heritage’ and ZIH-e1) and *R. occidentalis* (‘Bristol’ and Preston_2).

Table 1 summarizes the results of the amplification test. Of the 36 primer pairs tested that were designed from *R. idaeus* sequences, 25 pairs amplified a product, 19 of which produced a polymorphic product in *R. idaeus*. Of the 24 primer pairs designed from *R. occidentalis* sequences, 20 pairs amplified a product, 13 of which produced a polymorphic product in *R. occidentalis*. Of the 60 total primer pairs tested, 46 (76 %) produced amplification products that could be used to distinguish between the two species. In general, number and size range of alleles produced were similar between the two species. In terms of transferability, 22 of the 36 primer pairs (61 %) designed from *R. idaeus* sequence amplified a product in *R. occidentalis*, 18 (50 %) of which were polymorphic in *R. occidentalis*. Transferability from *R. occidentalis* to *R. idaeus* was demonstrated with 19 of the 24 primer pairs (79 %) amplifying a product of which 17 (71 %) detected polymorphisms in *R. idaeus*. These results indicate that markers that amplify a polymorphic product in highly-homozygous black raspberry are likely to amplify a polymorphic product in red raspberry, regardless of the sequence source.

**Table 1:** Summary of results for a subset of primer pairs designed for 60 expressed sequence tag (EST) loci derived from red raspberry (RI) and black raspberry (RO) sequences. Primer pairs were evaluated for the production of polymorphic PCR products and the ability to distinguish between the two species. Amplicon sizes are in base pairs (bp). Those primer pairs with unclear results are indicated as “unk”

	Polymorphic in Black Raspberry	Polymorphic in Red Raspberry	Number of alleles in Black Raspberry	Number of alleles in Red Raspberry	Amplicon size range Black Raspberry (bp)	Amplicon size range Red Raspberry (bp)	Distinguish between species?	Comments
RI_CHEa0001J04f	y	y	8	9	129–335	128–334	y	
RI_CHEa0001K23f	y	y	7	9	101–300	102–300	y	
RI_CHEa0001M05f	y	y	10	9	138–344	139–343	y	
RI_CHEa0001N07f	y	y	7	7	124–383	124–386	y	
RI_CHEa0002A10f	y	y	9	12	127–266	127–269	y	
RI_CHEa0002G14f	y	y	7	8	127–281	122–277	y	
RI_CHEa0002J02f	y	unk	3	2	130–233	174–182	y	
RI_CHEa0002K01f	y	y	18	14	117–395	117–392	y	
RI_CHEa0002L24f	y	y	8	8	112–264	113–265	y	
RI_CHEa0002N01f	y	y	3	4	171–372	135–292	y	
RI_CHEa0003H23f	y	y	11	10	117–321	117–298	y	
RI_CHEa0003N21f	y	y	10	13	131–295	117–295	y	
RI_CHEa0003O01f	y	y	22	19	108–393	108–387	y	
RI_CHEa0004B20f	y	y	7	6	180–297	191–332	y	
RI_CHEa0004H20f	y	y	17	15	110–390	110–385	y	
RI_CHEa0004L23f	y	y	10	11	112–403	112–383	y	
RI_CHEa0004P08f	y	y	5	6	132–153	131–154	y	
RI_CHEa0005M24f	y	y	11	13	179–402	176–395	y	
RO_CBEa0002O01f	y	y	6	9	110–330	110–334	y	
RO_CBEa0004M17f	y	n	4	2	111–331	111–322	y	Polymorphism in black raspberry needs validation
RO_CBEa0005H05f	y	unk	7	7	134–315	142–319	y	Inconsistent amplification for Heritage
RO_CBEa0005I06f	y	y	10	8	102–327	110–284	y	Polymorphism in black raspberry needs validation
RO_CBEa0006A02f	y	y	6	6	110–290	107–292	y	Poor amplification in one Bristol replicate
RO_CBEa0007C05f	y	y	7	12	110–329	109–332	y	Poor amplification in one Bristol replicate
RO_CBEa0007K08f	y	y	3	5	254–317	130–317	y	Inconsistent amplification in ZIH–e1
RO_CBEa0008E02f	y	y	13	12	115–415	117–415	y	
RO_CBEa0008O22f	y	y	5	5	120–290	122–279	y	Inconsistent amplification in Preston_2; only one replicate of ZIH–e1
RO_CBEa0009K12f	y	y	2	4	160–184	155–355	y	Polymorphism in black raspberry needs validation; inconsistent amplification in Heritage
RO_CBEa0009N10f	y	y	11	11	108–298	108–295	y	
RO_CBEa0010G06f	y	y	15	15	108–287	115–287	y	Poor amplification in one ZIH–e1 and one Bristol replicate
RO_CBEa0010M20f	y	y	16	14	115–415	115–415	y	
RI_CHEa0001H16f	n	n	1	4	283	103–286	y	Poor amplification for Bristol, Preston_2, and Heritage
RI_CHEa0003C04f	n	y	1	3	260	254–260	y	Poor amplification for Bristol and Preston_2
RI_CHEa0005E12f	n	n	1	1	278	278	n	
RI_CHEa0005K13f	n	n	1	1	277	277	n	
RI_CHEa0005P17f	n	y	2	3	226–256	226–308	y	
RO_CBEa0001B17f	n	y	2	2	153–160	157–248	y	One replicate of Preston_2 failed
RO_CBEa0003P15f	n	n	7	7	110–318	110–318	n	Poor amplification in one Preston_2 replicate
RO_CBEa0008G23f	n	y	5	6	107–219	107–269	y	
RI_CHEa0001C22f	unk	n	unk	1		151	unk	Poor amplification for Bristol, Preston_2, and ZIH–e1
RI_CHEa0002D18f	unk	unk	unk	unk	unk	unk	unk	Poor amplification for all samples
RI_CHEa0002G20f	unk	n	unk	1	unk	279	unk	Poor amplification for all samples
RI_CHEa0002H09f	unk	unk	unk	unk	unk	unk	unk	Poor amplification for all samples
RI_CHEa0002H15f	unk	unk	unk	unk	unk	unk	unk	Data for Bristol and Heritage only; only one replicate of Heritage amplified; poor amplification.
RI_CHEa0002L16f	unk	unk	unk	unk	unk	unk	unk	Poor amplification for all samples
RI_CHEa0003D14f	unk	n	3	3	172–201	172–201	n	Only one black raspberry replicate (Bristol) was successful; poor amplification for ZIH–e1
RI_CHEa0004B18f	unk	unk	unk	unk	unk	unk	unk	Poor amplification for all samples
RI_CHEa0004N08f	unk	unk	unk	unk	unk	unk	unk	Poor amplification for all samples
RI_CHEa0004P09f	unk	n	7	8	114–384	112–391	y	Only data for black raspberry is Bristol; poor amplification for ZIH–e1
RI_CHEa0005B17f	unk	unk	3	2	281–362	190, 281	y	Poor amplification for Bristol and Heritage.
RI_CHEa0005I04f	unk	unk	10	10	141–395	140–389	unk	Only one black raspberry replicate (Preston_2) was successful; poor amplification for ZIH-e1
RI_CHEa0005P15f	unk	unk	3	3	129–140	129–213	y	Only one red raspberry replicate (ZIH-e1) was successful; poor amplification for Bristol
RO_CBEa0001C08f	unk	unk	3	3	123–291	120–285	y	Both Bristol and one Preston_2 replicates failed; poor amplification for Heritage
RO_CBEa0001L10f	unk	y	14	12	115–298	122–298	y	One replicate of Bristol failed; inconsistent amplification for Preston_2
RO_CBEa0002K20f	unk	unk	5	8	140–315	138–315	y	Poor amplification in both Bristol replicates; inconsistent amplification for Preston_2, Heritage and ZIH-e1
RO_CBEa0002P20f	unk	unk	unk	unk	unk	unk	unk	One replicate of Bristol failed; poor amplification in second Bristol and one Heritage replicate
RO_CBEa0005J12f	unk	y	6	4	123–284	149–179	y	Only one black raspberry sample (Bristol) was successful
RO_CBEa0005J24f	unk	unk	6	7	162–485	159–486	y	Inconsistent amplification for all samples
RO_CBEa0005N17f	unk	y	6	7	110–290	109–293	y	Poor amplification in one Bristol replicate
RO_CBEa0006C18f	unk	y	2	6	133–252	133–256	y	Poor amplification in both Bristol replicate; inconsistent amplification for Preston_2

### Sequence functional characterization

The main reason for creating the *Rubus* libraries and sequence resources was for marker discovery; however, functional annotation of the sequences is a useful supplement for mapping efforts. Functional annotation allows investigators to target specific functional signatures of interest when testing molecular markers and allows the application of the sequences in a broader range of research questions. The functional information also provides a quality check for the library; we expect to see almost all sequences matching a model plant species and spanning a diversity of functions characteristic of leaf tissue. For this purpose, we chose to combine the transcripts from the two raspberry libraries into a single unigene set to provide the maximum amount of information about genes expressed in raspberry leaves and get the longest possible transcripts for searching and comparing to other genes. The combined raspberry unigene set has 418 contigs and 1671 singletons for a total of 2089 unigenes. The number of combined contigs was less than the sum of the contigs from the two datasets used for SSR identification, as identical contigs derived from both *Rubus* species were combined.

**Fig. 1:**
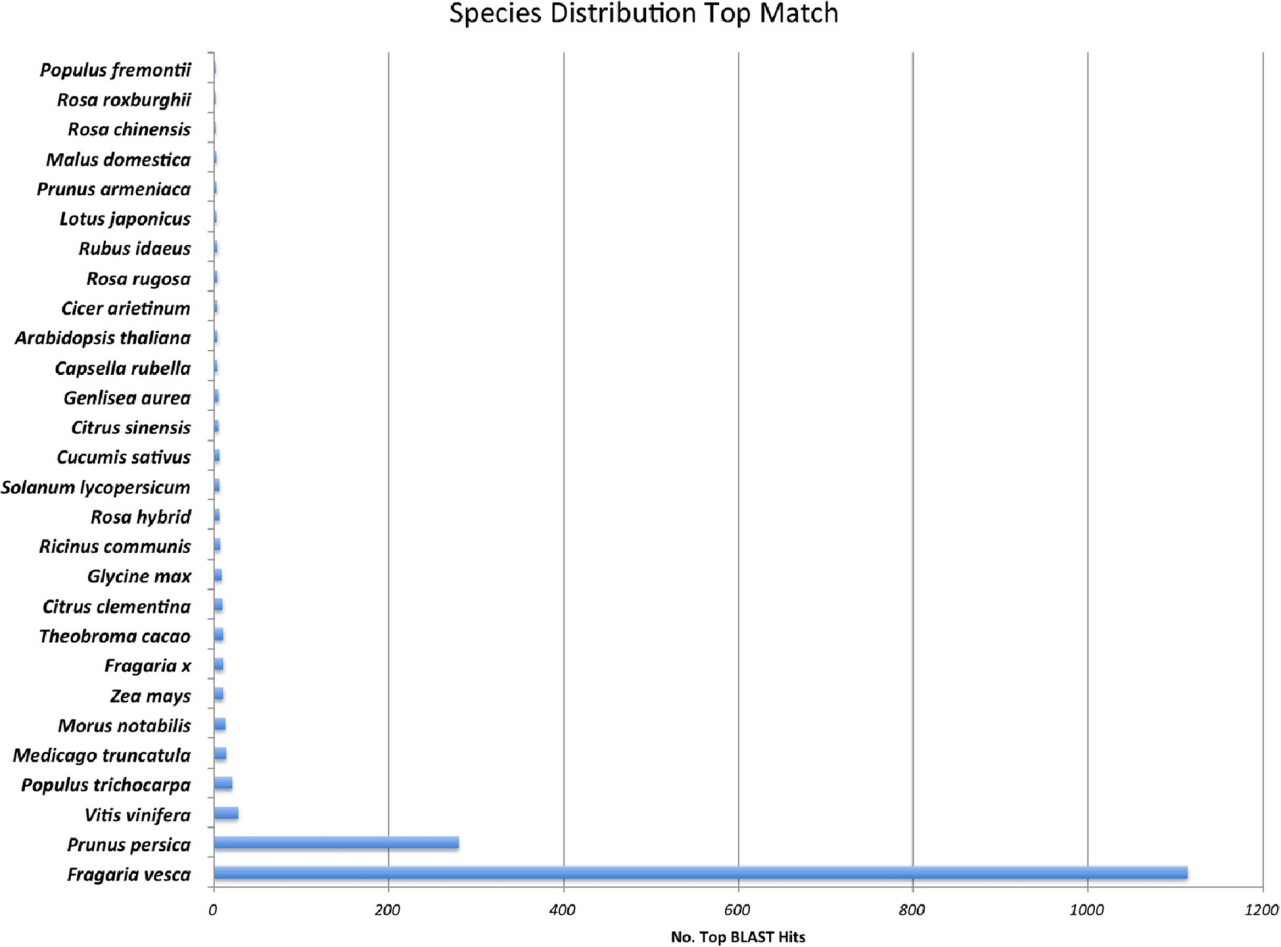
A basic local alignment search tool (BLAST) comparison of the 2145 combined black and red raspberry unigene set to the non-redundant (nr) protein database from the National Center for Biotechnology Information (NCBI). Results indicate that the majority of the unigenes aligned to genera in the rosid clade

A basic local alignment search tool (BLAST) [45] comparison of the 2089 unigenes to the non-redundant (nr) protein database from the NCBI [46] yielded matches for 1664 unigenes (80 %). Only six of these (0.003 %) had a best match to an organism outside of green plants. The majority, 1570 (94 %) had a best match to a plant in the rosid clade (Fig. 1). This confirms that the library has little, if any, contamination with microbes from either the sampling or laboratory procedures.

**Fig. 2:**
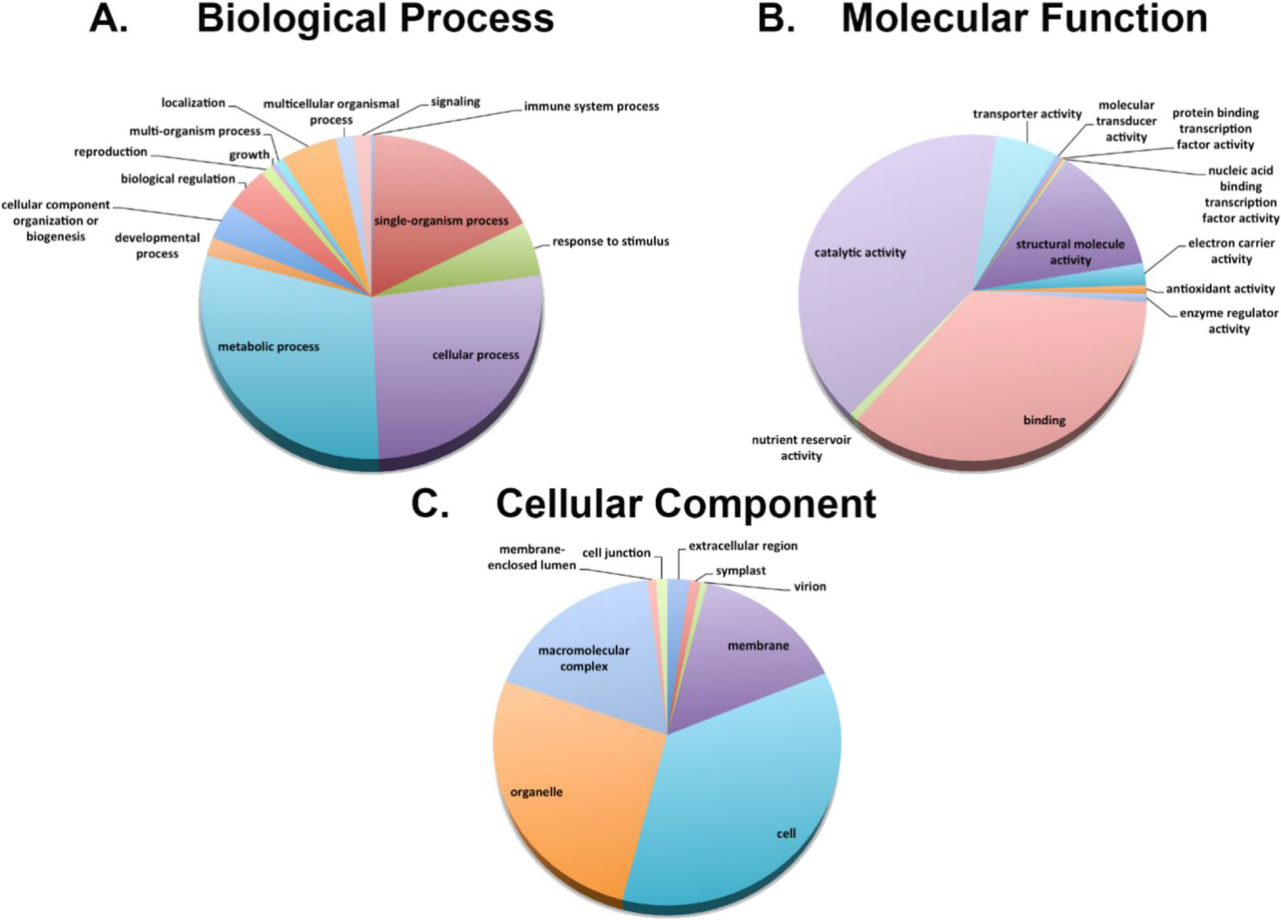
The unigene set was aligned to the Gene Ontology (GO) database [47] and classified according to the three basic categories: biological process, molecular function, and cellular component. The most abundant level 2 GO category was biological process with a total of 708 sequences associated with metabolic processes (211), cellular processes (187), and single organism processes (122). Other representative terms of biological process were response to stimulus (38), localization (38), and biological regulation (30) (Fig. 2a). GO assignments for the category molecular function totaled 366 sequences with functions for catalytic activity (148), binding (128), and structural molecule activity (47) (Fig. 2b). GO assignments for the category cellular component totaled 465 sequences assigned to cell part (164) and organelle (123) (Fig. 2c)

The unigene set was aligned to the Gene Ontology (GO) database [47] and classified according to the three basic categories: biological process, molecular function, and cellular component (Fig. 2). The most abundant sub-level two GO category was biological process with a total of 708 sequences associated with metabolic processes (211), cellular processes (187), and single organism processes (122). Other representative terms of biological process were response to stimulus (38), localization (38), and biological regulation (30) (Fig. 2a). GO assignments for the category molecular function totaled 366 sequences with functions for catalytic activity (148), binding (128), and structural molecule activity (47) (Fig. 2b). GO assignments for the category cellular component totaled 465 sequences assigned to cell part (164) and organelle (123) (Fig. 2c). A more detailed view of the GO sub-levels 3–5 reveals a significant fraction of genes related to metabolic processes such as macromolecule metabolism, organic substance metabolism, biosynthetic processes, and nitrogen/phosphorus metabolism (Additional file 3). Within the category molecular function, binding-related sub-categories such as cation binding, ion binding, and nucleoside binding were enriched. Finally, within the category cellular component, membrane, macromolecular complex, and symplast sub-categories were enriched (Additional file 3). Contig lengths ranged from 124 bp–1465 bp with an average length of 558 bp. To provide an example of functional diversity we aligned the ten longest unigenes to the GO database and identified a diversity of gene functions including heat shock, protease activity, and photosynthetic function (Additional file 4). All these annotations are reasonable for a set of genes from a plant leaf, and demonstrate the diversity of activities that were identified from a small set of ESTs.

**Fig. 3:**
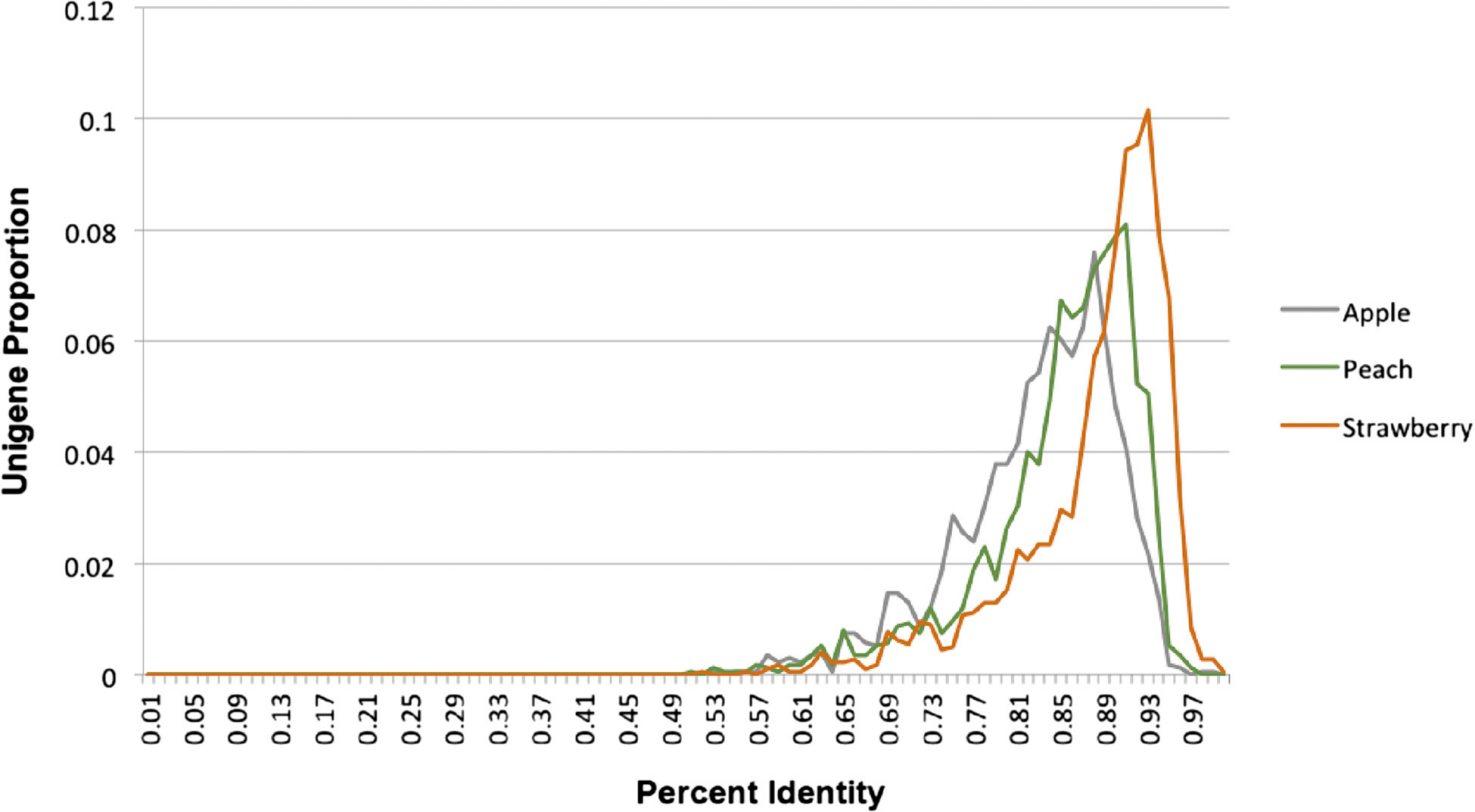
The distribution of percent sequence identities from alignments of raspberry unigenes to apple, peach, or strawberry genes. The greater similarity between raspberry and strawberry is a result of their close phylogenetic relationship relative to the other two crops

Reference genomes have been published from members of the Rosaceae: diploid strawberry (*Fragaria vesca* L.) [48], which is in the same subfamily (Rosoideae) as raspberry [49], double haploid peach (*Prunus persica* L.) [50], apple (*Malus* × *domestica* Borkh.) [51], European pear (*Pyrus communis* L.) [52], and Asian pear (*Pyrus bretschneideri* Rehd.) [53]. If enough sequence conservation exists between these genomes and raspberry, some of these new raspberry-derived markers and primers designed from polymorphic regions may be transferable to the other genera. The gene space in particular should be well conserved; therefore the raspberry unigenes were aligned to the gene sets from strawberry, peach, and apple to evaluate the actual sequence conservation. The best match for each unigene was re-aligned with a Smith-Waterman search [54] to obtain the best possible alignment. Considering all of the best alignments between raspberry and strawberry genes, 56.1 % of the alignments had greater than 90 % identity; when aligned to the peach genome, 29.7 % of the matches had a greater than 90 % identity; and for apple genes, 15.7 % of the matches had greater than 90 % sequence identity. Figure 3 illustrates this trend for percent identity across all alignments, demonstrating that the raspberry unigenes have an overall higher percent identity to strawberry than to the other two gene sets, which is consistent with their closer phylogenetic relationship.

## Conclusion

We have generated 121 and 257 EST-SSRs derived from leaf tissue of red raspberry (*R. idaeus*) and black raspberry (*R. occidentalis*) respectively. We have also designed 131 and 288 primer pairs for red and black raspberry, respectively. This resource constitutes a first step toward developing *Rubus*-specific, gene-derived markers that will facilitate the construction of linkage maps comprised of transferable markers for studying and manipulating important traits. The utility of some of these markers has been demonstrated already in the works of Dossett et al. 2010 [42] and Bushakra et al. 2012 [14], where some were used to evaluate genetic diversity among a wide selection of black raspberry genotypes and in genetic linkage map construction, respectively.

The advent of inexpensive next generation sequencing technologies has led to an increase in the use of SNP markers derived from high-throughput methods such as genotyping by sequencing (GBS) [55] and restriction site associated DNA (RAD) tags [56]. However, we argue that the long-utilized SSR is still the most effective and efficient marker type in certain circumstances. High-throughput sequencing costs are often reported as attractively low, but additional significant costs are associated with optimizing the restriction enzyme-based DNA preparations for a new species of interest, applying an appropriate informatics pipeline to manage the huge amount of sequence data, and finally to call the SNPs from an often “sparse” resulting data matrix [57, 58]. The same statistical power can be achieved with many fewer multiallelic SSRs than with biallelic SNPs derived from the complex GBS process. In the case of *Rubus* spp., where a reference genome is not yet available, the lack of key informatics poses an even more significant barrier to sequence-based SNP assays, such as the inability to align the SNPs to a reference, which requires additional work to assemble the sequencing reads. Also, specific to the *Rubus* spp. system, multiple species often are utilized and crossed in breeding programs. SSRs are significantly more likely than SNPs to transfer between species with little to no additional informatics investment. Considering the significant advantages, we selected SSRs as the best tool for straightforward yet effective genetic marker studies in *Rubus* species.

## Methods

### Plant material

Plants of ‘Heritage’ red raspberry and ‘Bristol’ black raspberry were purchased from Nourse Farms (Wately, Massachusetts, USA) and grown in pots in a greenhouse at Clemson University (Clemson, South Carolina, USA). Greenhouse conditions were 31.2 % relative humidity and 25 °C (76.7 °F). Approximately 5 g of young expanding leaf and meristem tissue from healthy plants was harvested from ‘Heritage’ and ‘Bristol’ on November 7, 2007 at approximately 10:00 a.m. EST, then immediately frozen in liquid nitrogen, and stored at −80 °C prior to RNA extraction. Leaf tissue from breeding selections ZIH-e1A, a red-fruited *R. idaeus*, and Preston_2, a yellow-fruited *R. occidentalis*, was kindly donated by Dr. Harry Swartz.

### cDNA library construction and sequencing

Total RNA was extracted using modifications to the methodologies of Meisel et al. [59]. Polyadenylated RNA was enriched using the Ambion® PolyA+ purist kit (Life Technologies, Grand Island, NY, USA) and was the substrate for cDNA synthesis. First- and second-strand synthesis was performed with the BD biosystems SMART® PCR cDNA synthesis kit (Clontech Laboratories, Inc.) and directionally cloned into the sfiA/B site of the vector pDNR-LIB (Clontech Laboratories, Inc.). A survey of the size of the insert in a subset of 48 clones, as assessed by resolving a polymerase chain reaction (PCR) product on 1 % agarose gels, revealed an average insert size of 750 bp. DNA isolation was carried out in 96-well format using standard alkaline lysis conditions [60]. DNA sequencing was performed with BigDye v3.1 (Applied Biosystems, Inc.) and raw trace data collected on an ABI 3730xl DNA analyzer (Applied Biosystems, Inc.).

### EST processing

The EST sequences were compared against the UniVec database from NCBI (ftp://ftp.ncbi.nih.gov/pub/UniVec/) to detect the presence of vector and adapter sequences. The program Cross_Match was implemented with the Consed package [61] and sequences quality trimmed of the vector and adapter sequences using the Lucy software [62]. Sequences with greater than 5 % ambiguous nucleotides (indicated by N) or fewer than 100 high quality bases (Phred score of ≥20) were discarded. The resulting high-quality cleaned ESTs were assembled into unigenes with the contig assembly program CAP3 [63] with empirically chosen parameters (−p 90 − d 60) to minimize assembly errors. The unigene set consists of the assembled contigs and the singletons output from CAP3.

A modified version (CUGISSR) of a Perl script SSRIT incorporated into the GDR tools [36, 64] was used to find perfect repeats meeting the following minimum requirements: 5 repeats of a 2 bp motif, 5 repeats of a 3 bp motif, 4 repeats of a 4 bp motif, or 3 repeats of a 5 bp motif. Primer sequences for the identified SSRs were generated using the Primer3 program [65]. To establish the SSR positions in relation to coding region, putative open reading frames (ORFs) were identified with the software FLIP [66]. All of these data are available in a Microsoft® Excel file through the Supplemental Materials.

The two sets of raspberry ESTs were combined into a single unigene with the CAP3 software program with empirically chosen parameters (−p 90 − d 60) prior to being functionally characterized. Homology searches using BLAST [45] were performed with an E-value cutoff of 1^e-6^ against the NCBI nr protein database. To assign GO terms, the software Blast2GO [67] was run utilizing the NCBI nr results. The GO results and discussion in this publication refer to the functional results from the combined unigene.

Further comparisons of the combined *Rubus* sequences to the wider Rosaceae taxa were completed by performing a BLAST search to the protein coding sequences (CDS features) associated with three recently published whole genome sequences: *Fragaria vesca* [48], *Prunus persica* [50], and *Malus* × *domestica* [51]. All three sets were downloaded from the Genome Database for Rosaceae (http://www.rosaceae.org/). The hybrid *Rubus* gene models were chosen for comparison to *Fragaria vesca*. To get the best possible contiguous alignment, each raspberry unigene was compared to its best CDS match in each of the three genomes with SSearch [68], a software program that performs a rigorous Smith-Waterman alignment.

### PCR test of a subset of SSR primer pairs

A subset of 36 primer pairs from the 131 designed to test the 98 individual unigenes identified in red raspberry, and 24 primer pairs from the 288 designed to test the 207 unigenes identified in black raspberry were identified using random sorting of the source sequences in a Microsoft® Excel file and assessed in PCR. Primer pairs were evaluated for PCR amplification, production of polymorphic products and transferability between species. Amplification was tested with two genotypes each of *R. idaeus* (‘Heritage’ and ZIH-e1A) and *R. occidentalis* (‘Bristol’ and breeding selection Preston_2). DNA extraction, polymerase chain reactions (PCR) and sizing of PCR products followed Stafne et al. [69].

PCR products were visualized using an ABI 3730 Genetic Analyzer (Applied Biosystems, Inc.) and analyzed using ABI GeneMapper software v4.0.

## Additional files

Additional file 1:
**NCBI accession, locus name, and details of SSR, primer design and DNA sequence for red raspberry (R. idaeus).** Highlight indicates those loci tested in R. idaeus and R. occidentalis genotypes with results shown in manuscript Table 1. (XLSX 48 kb) Additional file 2:
**NCBI accession, locus name, and details of SSR, primer design and DNA sequence for black raspberry (R. occidentalis).** Highlight indicates those loci tested in R. idaeus and R. occidentalis genotypes with results shown in manuscript Table 1. (XLSX 93 kb) Additional file 3:
**Gene ontology term distribution for the categories Biological Process, Molecular Function, and Cellular Component.** (XLSX 12 kb) Additional file 4:
**Top ten longest unigenes aligned to the Gene Ontology database with BLAST results.** (XLSX 10 kb)
